# Paramedics in an emerging role; a reflexive thematic analysis of barriers and enablers to primary care innovation through a community of practice

**DOI:** 10.1186/s12875-026-03426-y

**Published:** 2026-06-23

**Authors:** R Sharples, L Hemming, R Hardman, E Spelten, K Sutherland, G. Agarwal, L Reynolds

**Affiliations:** 1 Violet Vines Marshman Centre for Rural Health Research, Rural Health School, La Trobe University, Bendigo, Victoria Australia; 2 Sunraysia Community Health Services, Mildura, Victoria Australia; 3 Australian Catholic University, Melbourne, Victoria Australia; 4 Safer Care Victoria, Melbourne, Victoria Australia; 5 Department of Family Medicine, McMaster University, Hamilton, Ontario Canada

**Keywords:** Community paramedicine, Primary health care, Rural and remote health, Community of practice, Workforce development, Chronic disease management

## Abstract

**Background:**

Community paramedicine is an emerging model of care that focuses on preventative and rehabilitative health and improves equitable access to primary care. Four community health services in rural and regional Victoria, Australia, implemented the evidence-based Canadian CP@clinic (Community Paramedicine at Clinic) program. The paramedic-led program delivers free drop-in clinics with chronic disease screening, onward referrals, care navigation and health education.

**Methods:**

We conducted a reflexive thematic analysis of de-identified monthly meeting records of 13 Community of Practice (CoP) meetings. Analysis specifically focussed on the barriers and enablers of program delivery and on how the CoP supported CP@clinic program administrators, researchers and community paramedics.

**Results:**

CP@clinic implementation in rural, regional and remote Australia was shaped by four interrelated themes: workforce development, community engagement, data collection and program sustainability. Barriers centred on role ambiguity and transition from emergency care, the time intensity of trust-building within communities and referral navigation, administrative burden compounded by digital infrastructure constraints and persistent funding and workforce instability. Within these dynamics the CoP emerged as a crucial implementation mechanism that enabled both individual practitioners and organisations to navigate uncertainties, build capacity and maintain program adherence.

**Conclusion:**

CP@clinic can take root in rural, regional and remote Australia but only if the conditions are right. The CoP acted as the engine room for implementation accelerating role transition, local adaptation and practical problem solving. Yet persistent barriers - role ambiguity, time-intensive trust building, heavy data demands in low-connectivity settings and funding instability - continue to stall momentum. To move from a promising pilot to durable system change, the CP@clinic program needs CoP-enabled learning along with structural investment in training, digital infrastructure, evaluation support and long-term funding.

**Supplementary Information:**

The online version contains supplementary material available at 10.1186/s12875-026-03426-y.

## Background

Community paramedicine is an emerging model of care that extends the role of registered paramedics beyond emergency response to deliver timely, community based primary care and reduce avoidable conveyance to emergency departments thereby mitigating the risks associated with delayed or fragmented care [1–3]. As an applied primary care strategy, community paramedics use their existing clinical skills supported by additional training to provide safe, low-acuity care in people’s usual place of residence or other community settings, rather than defaulting to hospital-based services [4, 5].

### Community paramedicine and equity policy implications

Community paramedicine harnesses an already qualified workforce to deliver accessible, first-contact primary health care for socially and geographically marginalised populations aligning with World Health Organization equity principles [6–8]. By targeting people with poor access to general practitioners or who rely on emergency departments for primary care-type conditions, community paramedicine aims to narrow health inequities and strengthen community-based management of low acuity and chronic conditions [4, 9]. Yet paramedics remain largely excluded from Australia’s Primary Health Care 10 Year Plan, which confines them to state-based ambulance models, missing an opportunity to embed this workforce in equity focused reform despite evidence of avoidable lower urgency emergency department presentations [10–12]. Given the absence of visibility of paramedicine in health policy in non-jurisdictional settings, funding support for innovative program implementation represents a key external barrier to expanding scopes of practice in primary health care, particularly in rural areas.

### The CP@clinic model

Developed at McMaster University in Ontario, Canada, the Community Paramedicine at Clinic (CP@clinic) programme is a rigorously researched, evidence based and innovative model that has been implemented in diverse community health settings in Canada and Australia [4, 9]. In CP@clinic, registered paramedics are trained to deliver free, drop-in chronic disease assessment clinics in accessible locations such as social housing common rooms and community centres targeting predominantly older adults at risk of, or living with, multiple chronic conditions and who are high users of emergency services [4, 13]. Community paramedics conduct regular clinics that include structured health assessments (for example blood pressure, diabetes and falls risk screening), tailored health education and referrals to primary care and community services with feedback to family physicians to support continuity of care [9, 14]. Strong evidence, including a cluster randomised trial and economic evaluations, shows that CP@clinic reduces chronic disease risk factors, improves frailty related-outcomes, lowers emergency call volumes and is cost-effective for clients, the paramedic workforce, communities and the wider health system through reduced ambulance callouts and emergency department presentations [13, 15]. In rural and regional Victoria, Australia, CP@clinic therefore functions as an innovative pragmatic solution that leverages an existing available workforce to extend primary health care access in communities with longstanding shortages of general practitioners or allied health practitioners.

### Communities of practice

To support implementation, communities of practice (CoPs) can provide a structured yet flexible mechanism for workforce learning and service change. CoPs are defined by shared interest, practice and community underpinned by a social theory of learning that emphasises participation and identity formation and in healthcare they are frequently used to introduce and sustain new models of care by enabling clinicians to share tacit knowledge, problem solve and build collective competence across organisational boundaries [16–20].

The current study examines the barriers and enablers to the implementation of the Canadian CP@clinic program in rural and regional Victoria through the lens of a CoP. It also explores how the CoP supported implementation and knowledge sharing for program administrators, community paramedics and researchers. Implementation of the CP@clinic program commenced with a 10-month pilot in north-west Victoria in 2023, followed by the establishment of a CoP in January 2024 to support program expansion across additional rural Victorian sites. In this study, a CoP was established as an implementation and learning network for the CP@clinic program administrators, community paramedics and local and international research team members. Given the novelty of community paramedicine in Australian primary health care settings and the lack of policy governance for this workforce, the CoP was purposefully designed to provide a facilitated engagement opportunity in which participants could share emerging practice experiences, collectively problem-solve implementation challenges, develop shared understandings of the community paramedic role and support the contextual adaptation of CP@clinic across diverse rural community health settings.

## Methods

The aim of this study was to examine implementation of the CP@clinic program in rural and regional Australian communities through the lens of a CoP. Specifically, we sought to: [1] identify the barriers and enablers to CP@clinic program implementation as described in CoP meetings, and [2] explore how the CoP supported community paramedics, researchers and program administrators in program implementation to deliver this innovative model of care.

The following section is structured using the Reflexive Thematic Analysis Reporting Guidelines (RTARG) [21] with further details in the supplementary materials file related to method and analysis.

### Setting and participating services

**Fig. 1:**
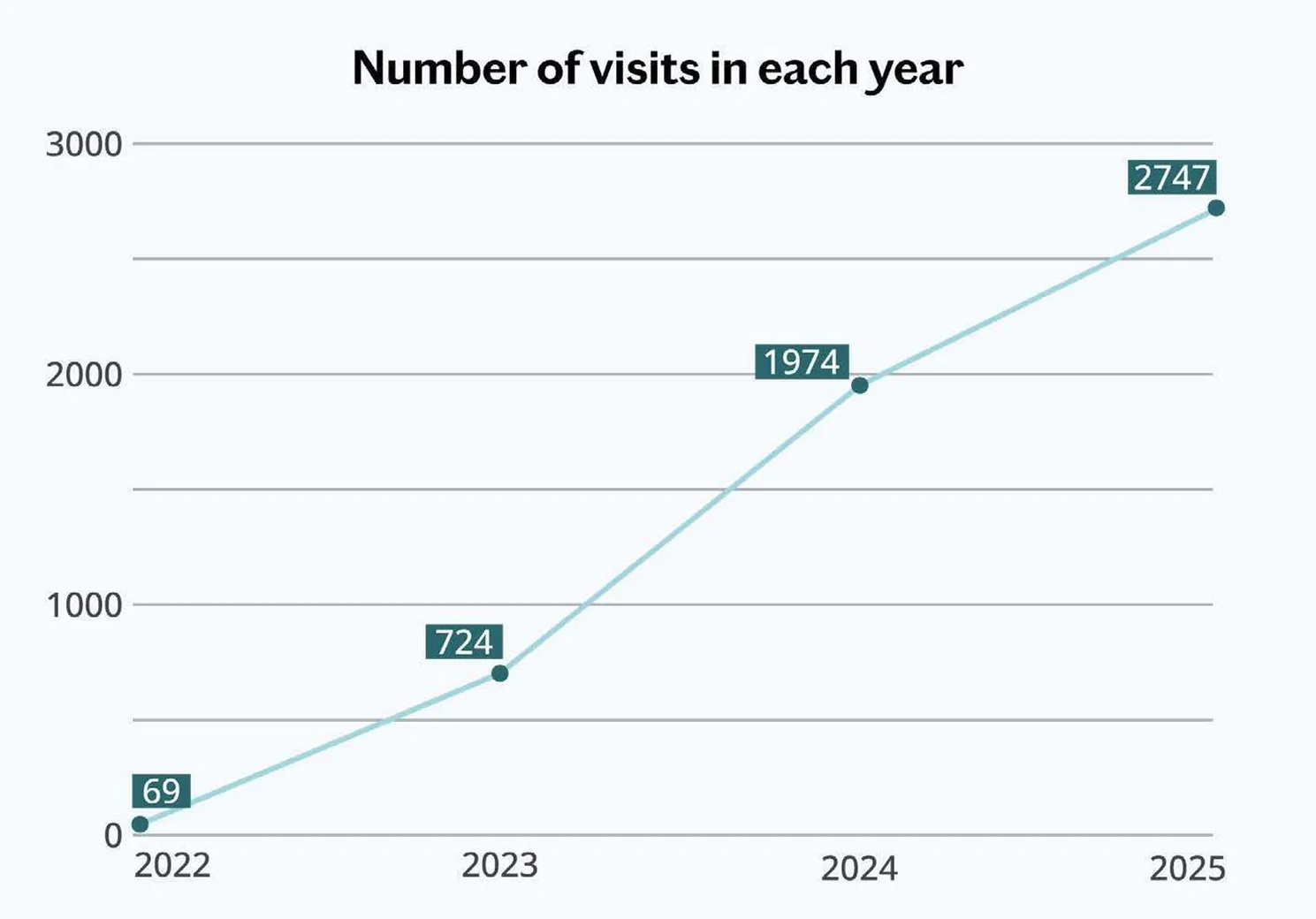
Number of individual CP@clinic client visits 2022–2025

Program administrators and community paramedics from four [4] Victorian rural and regional community health services participated in the CoP. These services operate across Modified Monash Model (MMM) 3–5 regions which are characterised by relative social disadvantage, ageing populations and ‘thin markets’ with limited access to health practitioners [22]. Below is a figure of the cumulative numbers of clinic attendances across the four [4] sites and total in all regions to demonstrate the scaled implementation - see fig. 1.

### Funding and program initiation

Implementation of the CP@clinic program commenced with a 10-month pilot in north-west Victoria in 2023 [9]. Given the positive results from the feasibility pilot, in 2024, as part of an Australian Department of Health and Ageing Innovative Model of Care (IMOC) grant, additional services joined in the scaled implementation and evaluation of the CP@clinic program.

### Participants/data items

Participants in this study were the stakeholders actively involved in the implementation of the CP@clinic program sites across Victoria, Australia. These included program administrators, community paramedics and researchers (see Table 1) who comprised the membership of the CoP since its inception and scaled implementation. CoP meeting records documented themed and focussed discussions relating to program implementation; challenges relating to consumer engagement, funding and integration with other services. Clients and other stakeholders (such as general practitioners, policy makers, funders) did not participate in the meetings and therefore not directly represented in the meeting records. Contributions were documented as de-identified notes without attribution to individuals however roles were noted to enable comparison.

**Table 1:** Participant characteristics by role

Participant Role	No of Participants (*n*)	Description
Community Paramedics	13	Delivered CP@clinic services in rural communities
Program Administrators	6	Responsible for logistics, funding and oversight
Researcher team	9	Involved in program design, evaluation and CoP facilitation.
Total	27	

### Dataset generation

Commencing in early 2024 and throughout 2025, CP@clinic program administrators, researchers and community paramedics met online via Zoom for 1-hour monthly to discuss their experiences. Each of the CoPs focussed on a topic/issue as identified [by last author (LA), third author (3A) and fourth author (4A)] that formed a loose agenda that allowed for facilitated free-flowing discussion (see Supplementary Table 1). Participants engaged regularly in reflexive dialogue, co-learning and problem-solving through the CoP structure. Meetings were chaired by LA, with attendance varied across meetings, which increased over time due to additional programs joining (see Supplementary Table 2).

With ethics approval granted [LTU HREC 22295], participants in this study consented to their participation with data collection. CoP meetings were video [23] recorded which enabled for transcription, summarisation and distribution after meetings to members by FA or 3A. These written meeting records served as the primary record of a summarised discussion on the focus areas. As such, the meeting records did not capture verbatim discussion attributable to specific individuals; rather, they reflected processes of shared meaning making within the CoP. These written meeting records formed 13 data sources between March 2024 and April 2025 which were deidentified.

### Data analysis

A reflexive thematic analysis (rTA) was conducted to address the study aims and research questions. This study adopted a qualitative methodology. Specifically, a ‘Big Q’ approach [24] was taken to address methodological congruence with the research question. This approach involved the researchers as active participants in the construction of knowledge with ongoing reflection as a circular process [21]. This ensured that knowledge was viewed as contextual, evolving and co-constructed through the analysis process [25] and drew on the ‘knowingness’ of the researchers given they are registered paramedics (FA and LA) who undertook the rTA and were able to acknowledge the complexity and situatedness of the themes as they were discovered [25].

FA and LA conducted an inductive, primarily semantic analysis. Records were segmented into discrete ‘mentions’ (sentences/bullet points/idea units) (see Supplementary Table 3). Then FA and LA looked for patterns of shared meaning across the revised codes and combined codes with similar meaning together. Codes were generated from the data and iteratively refined into themes. Mentions could be coded to multiple themes (see Supplementary Table 4).

Themes were organised into higher-order groupings aligned with implementation-focused domains, as well as groups that had a deeper shared meaning. After reviewing and refining the themes, FA and LA defined and named themes. In this step, the researchers provided clear definitions and descriptions for each of their final themes (see Supplementary Table 6).

This analysis was completed collaboratively (FA and LA), across four meetings to facilitate discussion (see Supplementary Table 5).

In keeping with reflexive thematic analysis approach, coding and theme development were treated as iterative, interpretive processes rather than fixed linear procedures. To support rigour and trustworthiness, FA and LA met regularly to review codes, refine thematic boundaries and interrogate alternative meaning drawing on their paramedicine expertise and reflecting on their positionality to shape the analysis. Analytic decisions were documented (see Supplementary Table 5) and emerging themes checked against the full dataset to ensure coherence and richness rather than frequency alone.

## Results

**Fig. 2:**
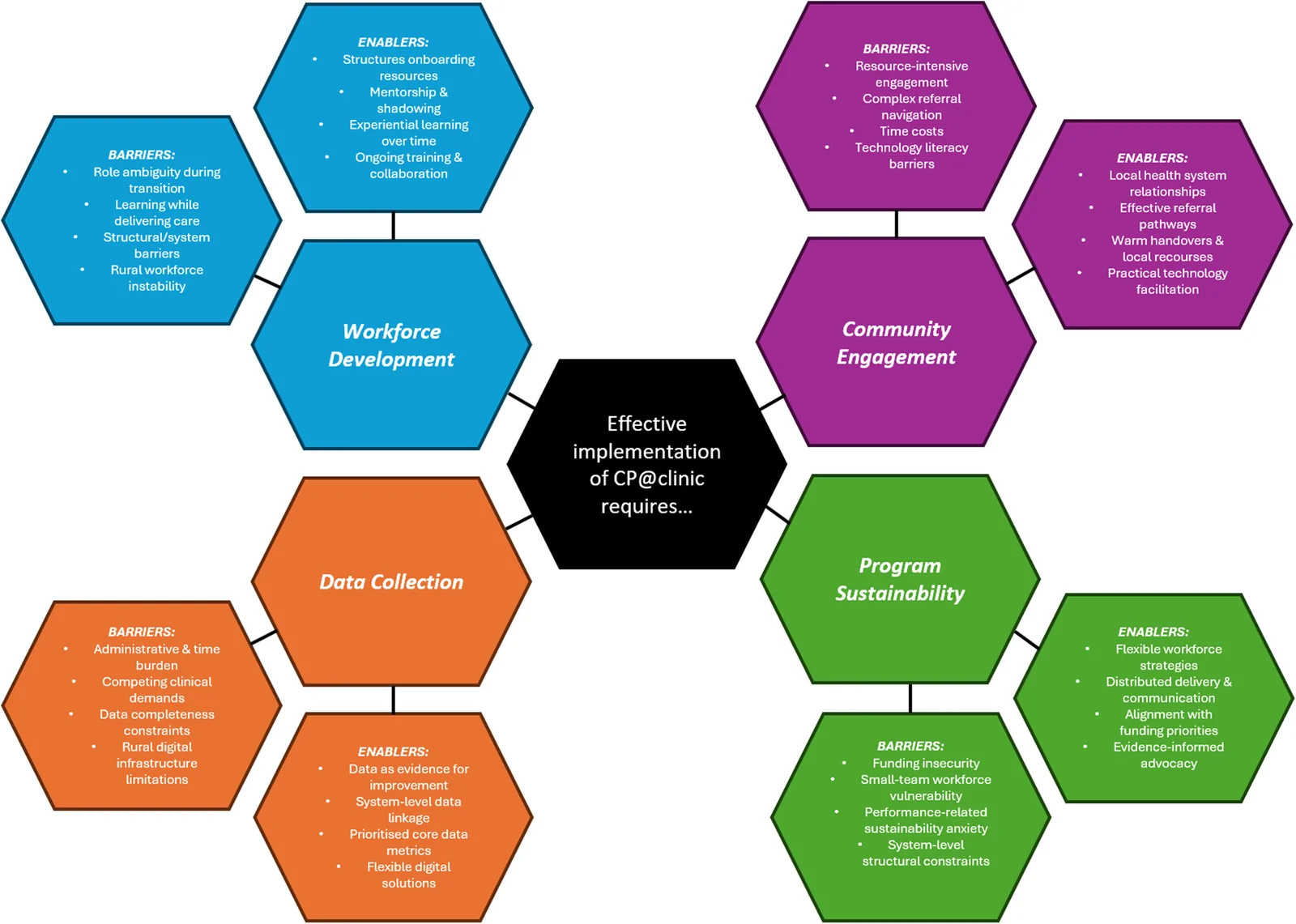
Conceptual map of barriers and enablers across four themes

rTA of CoP meeting records identified four interrelated themes that describe both the barriers and enablers shaping CP@clinic program implementation in rural, regional and remote Australia. Themes were closely interconnected (see Supplementary Fig. 1). For example the relationship-based approach that supported community engagement often required time and multiple visits, which in turn adversely affected the timing and completeness of data collection. Figure 2 (below) presents a conceptual map of the barriers and enablers identified across each of the four themes, illustrating how these factors shaped CP@clinic implementation through the lens of the CoP.

### Theme 1: Workforce development

#### Barriers

Workforce development *(see Supplemental Note N1)* was repeatedly described as challenging due to role transition and role clarity, particularly by paramedics who had moved from an emergency ambulance setting to preventative, community-based primary care practice. In Meeting #12 it was reported that *‘…the first year was a steep learning curve during the initial months’* emphasising the complexity of balancing timely assessments and database requirements alongside clinical responsibilities. Implementation was constrained by uncertainty about what the role encompassed, variability in expectations across settings and reliance on paramedics learning the model while simultaneously delivering clinics and establishing workflows. Participants described their experience of *‘…stepping into the program without fully understanding it’* and the challenges of *‘…balancing training with clinic responsibilities’ *(Meeting #12)*.* Client recruitment and retention were an ongoing challenge in rural and remote contexts, compounded by the need to demonstrate value to prospective staff and stakeholders while services were still emerging.

Structural constraints - such as broader workforce and system conditions (e.g. regulatory/industrial and education/technology contexts) - were also understood to shape workforce readiness and stability. Workforce challenges were further compounded by limited formalised training pathways and structural supports. Participants identified *‘…unfamiliarity with the role outside of the ambulance service*,* lack of professional development and no defined salary structure’* as barriers to workplace stability (Meeting #1). Participants also noted that learning extended beyond clinical skills. In Meeting #1 participants *‘…highlighted the learning opportunities in health coaching and navigating aged care’* identifying these as new areas of practice for paramedics. In Meeting #2 participants reported exposure to a range of referral pathways and services that had not been encounted in their prior jurisdictional ambulance service roles before commencing in their community paramedic roles.

#### Enablers

The CoP identified enablers that supported workforce development. These included structured onboarding supports such as training guides and prompts for assessments, as well as recognition that practice became more efficient with experience. Workforce transition was facilitated through informal mentorship and shadowing which were positioned as particularly important for new teams to gain confidence and adopt consistent approaches. In addition, ongoing training and collaboration - such as completing formal training and scheduling follow-up training sessions - were used to support expansion of community paramedics to new sites. Participants described training efforts that were actively underway including community paramedics *‘…completing McMaster training and scheduling additional sessions’* alongside opportunities for shadowing and mentoring (Meeting #9).

Participants emphasised the importance of collaboration and peer learning in overcoming early challenges. Participants noted ‘…*the importance of collaboration in overcoming challenges’* particularly when managing assessments and database requirements. Others described learning from experience and from peers (Meeting #12). Whereas it was noted that despite early challenges in managing clinics and time pressures, ‘*…the value of learning from experience and the support form peers’* was increasingly recognised as *‘…ongoing learning’* as they continued to refine their methods over time (Meeting #13).

The CoP was repeatedly identified as a safe space that supported reflection and professional growth. Workplace safety was discussed and *‘…the value of the Community of Practice as a space for reflection and peer support*,’ was highlighted, particularly in the absence of typical ‘ambulance health’ clinical supervision structures (Meeting #4). Mentorship was viewed as central to supporting paramedics transitioning from ‘ambulance health’ to community paramedic roles and promoting best practice, with participants reflecting on the importance of learning from existing staff regarding clinic operations, patient interactions and data collection.

### Theme 2: Community engagement

#### Barriers

Community engagement *(see Supplemental Note N2)* was described as essential to CP@clinic implementation yet time-intensive and highly dependent on local context. Engagement challenges included low awareness of the program, variable consumer needs and barriers related to technology and trust-building. As detailed in the meeting record, *‘…success can be defined differently across stakeholders*,* such as the healthcare system*,* community health organisations and consumers themselves’* (Meeting #9). 

Implementers noted that navigating referral pathways required learning new skills and working through internal community health processes with additional time required to ensure coordination across services. A key success in navigating referral pathways was identified as relationship building which was challenging as it required‘…*building new skills in the referral process*’ and ‘*navigating internal systems and processes relating to community health services*’ (Meeting #2). Time costs were particularly visible where warm handovers were needed to improve continuity across settings. In this context, warm handovers referred to facilitated health professional-to-health professional transfer of care or referrals. This could be participating in telehealth calls or coordinating the dispensing of community pharmacy medication as opposed to providing written information or contact details.

Engaging consumers was also shaped by local context including barriers related to technology literacy that could limit participation and require additional facilitation. Meaningful consumer engagement was acknowledged as important however ‘…*barriers to this*,* especially with consumers with low technology literacy*’ were also identified (Meeting #9). Building trust with clients was viewed as a factor for workplace safety as *‘… a key component of client safety and satisfaction*‘ (Meeting #4). For example, clients may be unwilling to engage in assessments such as height, weight and waist circumference measurements and it *‘…takes a few visits to develop the trust needed for that’* (Meeting #3). Non-technical paramedic skills were noted as important, particularly listening and rapport building, as participants noted the *‘importance of listening to clients to build trust and encourage self-management’ *and* ‘the value of taking time to listen to clients to build trust’* (Meeting #3).

#### Enablers

A consistent enabler was relationship-building across the local health system, including with general practitioners, emergency departments and community health services. Participants emphasised that implementation success depended on these relationships to create workable pathways and reduce delays for clients. CoP discussions also highlighted practical strategies to support engagement: investing time in understanding local services, using warm handovers to strengthen connections and developing local directories of resources (including health literacy materials), while recognising this required maintenance over time. As the program scaled, new implementers attending the CoP in Meeting #9 noted that the program ‘*…has multiple locations throughout its region’* while in Meeting #11 one participant reported ‘*…targeting communities affected by bushfires.’* These examples highlighted how *‘…many patient participants in the program are disengaged from traditional healthcare*,* but the program is successfully re-engaging them through accessible services and additional support like food relief and community meals’* (Meeting #7).

Community engagement requires program implementers to be innovative with practical strategies, adapt approaches and build local partnerships (Meeting #9). Participants described service development through collaboration with local organisations, noting that ‘discussions are underway with service providers such as ***Health to host clients in their facilities,* with a focus on existing sites and additional locations based on community needs.’* Workforce and service planning were similarly framed around community need, with meeting notes (#4) revealing that *‘the selected recruit will work alongside community health nurses, initially targeting areas identified as underserved with significant healthcare needs.’*

To address technology barriers in consumer participation, implementers proposed pragmatic supports such as helping clients set up their devices or facilitating telehealth appointments on-site, where clients attended the clinic and community paramedics assisted them to connect with other health practitioners via phone or video, framing these as workable adaptations to local needs.

### Theme 3: Data collection

#### Barriers

Participants repeatedly identified data collection *(see Supplemental Note N3)* as a practical barrier during CP@clinic implementation due to administrative burden, competing clinical demands and limits in digital infrastructure. These included barriers such as *‘… issues with data entry systems and the steep learning curve for new clinics’* while gaining *‘… efficiency*,* particularly for new sites’* (Meeting #11).

Implementers described challenges in their patient assessment while inputting data given that they have structured questioning as part of standardised assessments, creating tension between client-centred interaction and documentation.

Participants also highlighted difficulties related to data systems and recording practices across platforms. It was noted that information was sometimes captured in local electronic medical records rather than the CP@clinic database, raising concerns that important referral and engagement activities were not consistently visible for evaluation purposes. As one participant stated, it was ‘…*really important to record that info in the CP@clinic risk factor discussion to show that people have been connected and engaged*' (Meeting #6). Trust building with clients was also described as affecting data collection, with notes indicating that ‘*building trust makes data gathering easier*’ particularly for measures such as height, ‘*weight and waist circumference*,’ which ‘*can be challenging for some clients*’ (Meeting #3).

Infrastructure issues were especially common in the rural delivery context. Poor internet connectivity led to paper-based workarounds and delayed data entry, increasing workload and risking inconsistency.

#### Enablers

Despite these constraints data collection was also framed as an enabler when positioned as evidence for improvement and sustainability of the program. Implementers described the importance of data integrity and database development to support decision-making and quality assurance. The CoP facilitated discussions to not only identify challenges but to share approaches and practical workarounds that supported fidelity to program goals while reducing burden. Drawing on the Canadian program administrators attending the CoP, participants found reassurance in the observation that ‘… *each person might have a slightly different approach to the assessments*,* but with experience*,* the process becomes quicker and more efficient’* (Meeting #1). Reflecting in and on practice was also noted as important, particularly when teaching that ‘…*a few key prompts could guide the clinical approach and make the process more straightforward’* (Meeting #1).

The CoP played a key role in identifying data-related challenges and supporting collective problem solving. Participants shared practical strategies for improving efficiency and consistency including refining assessment processes over time. To make data capture feasible, the CoP discussed creating a hierarchy of essential data points so clinicians could prioritise core metrics when capacity was limited, to reduce unnecessary administrative load. Digital solutions were also treated as enabling, adapting the database to show incomplete forms, developing offline functionality with later synchronisation and resourcing connectivity improvements (e.g. modems).

The CoP also supported shared understanding of the purpose of data collection. Participants discussed how community paramedics could prioritise client engagement by allowing *‘data collection [to happen] in the background*' (Meeting #1), while still capturing essential information. Several participants reinforced the strategic value of data, noting that ‘*the data gathered would not only demonstrate effectiveness but also support strategic arguments to secure funding for expanding paramedics’ roles in primary care*' (Meeting #5). Linking community health data to larger state and national databases while maintaining privacy was also discussed as a strategy to strengthen evidence of program impact.

System-level evaluation was strengthened by planned data linkage across emergency departments, hospital admission and ambulance datasets to demonstrate program impact beyond the clinic setting.

### Theme 4: Program sustainability

#### Barriers

Program sustainability concerns were prominent *(see Supplemental Note N4)* and closely connected to funding uncertainty, workforce fragility and perceived responsibility for ongoing delivery. Participants identified how program sustainability relies on ‘…*funding certainty and longer-term assurance to continue building the program’s momentum’* (Meeting #1). Funding cessation was lamented as being contingent on measuring success and participants ‘….*worry that if the service isn’t ‘successful’ then funding will be discontinued and ultimately clients will be left without help’* (Meeting #5). Barriers such as funding were identified in ‘…*the national scope of practice review workshop and discussed barriers such as legislation*,* employer practices*,* funding*,* education and technology’* (Meeting #1).

Implementers emphasised the need for longer-term funding assurance to maintain momentum. At the service level, sustainability pressures were experienced as heightened responsibility in small teams including anxiety that fluctuations in reported outcomes or performance indicators could be interpreted as reduced success, threatening funding and leaving clients without adequate support. Broader system constraints - legislation, employer practices, funding arrangements, education and technology - were also discussed as factors shaping the program’s longer-term embedment.

#### Enablers

Enablers for sustaining program implementation focussed on maintaining continuity, building a persuasive evidence base and distributing workload. Participants emphasised ‘*the value of CoP as a space for reflection and peer support*,’ particularly in navigating uncertainty related to funding, workforce turnover and system constraints (Meeting #4). Services leveraged staffing strategies such as casual coverage and cross-training within community health services to reduce vulnerability to leave and staff turnover. Communication processes (e.g. use of organisational communications teams and direct messaging to repeat clients) were described as practical mechanisms to manage clinic cancellations or schedule changes and maintain engagement.

Sustainability was also enabled through aligning program reporting with funder and policymaker priorities including the use of healthcare utilisation outcomes to demonstrate system benefit and strengthen the case for ongoing investment. This strategic orientation positioned evaluation and advocacy as integral to implementation rather than an ‘add-on,’ particularly in rural contexts where service continuity was perceived as both fragile and consequential.

Participants further linked sustainability to visibility and advocacy. Activities such as conference presentations and collaboration with professional bodies were discussed as mechanisms for strengthening the program’s profile. Participants noted that ‘*success stories and patient case studies play an important role in making a compelling case for policy makers and funders*,’ reinforcing the importance of documenting and communicating program outcomes (Meeting #11). The CoP was positioned as central to maintaining momentum, coordinating advocacy efforts and supporting the long-term integration of community paramedicine within the broader healthcare system.

## Discussion

The aim of this study was to identify the barriers and enablers to implementing the CP@clinic program in regional and rural Australian communities drawing on insights from CoP meetings that supported community paramedics, researchers and program administrators in delivering this innovative model of care. In addition, we explored the role of the CoP in supporting implementation. rTA of 13 CoP meetings revealed four interrelated themes - workforce development, community engagement, data collection and sustaining program implementation. These themes highlight the challenges and opportunities involved in translating CP@clinic into diverse rural Australian contexts. The findings demonstrate that implementation is not merely a technical process but a relational and interpretive one, requiring role clarity, local trust, adaptive systems and sustained professional support with the CoP emerging as a mechanism for navigating uncertainty, building capacity and maintaining program integrity.

### The interrelatedness of enablers and barriers through the CoP lens

Across all themes, enablers and barriers were closely interrelated rather than discrete. While community engagement improved through shared learning, it remained time-intensive and uneven with some communities initially hesitant to engage with a paramedic-led preventative health model. This reflects the ongoing work required to establish legitimacy and acceptability across multiple audiences, not solely within clinical teams [26, 27]. Data collection most clearly illustrated this dynamic. It was repeatedly identified as both a barrier to feasibility and a key enabler of program legitimacy and sustainability. Administrative burden, competing clinical priorities and limited digital infrastructure threatened engagement and risked detracting from the relational care central to CP@clinic.

These four themes can also be understood in relation to the various properties of creating a CoP [17, 18]. Theme 1 (workforce development) aligns with Wenger’s concept of ‘mutual engagement’ and ‘learning as social participation’ as participants collectively negotiated new roles, responsibilities and ways of practicing. Theme 2 (community engagement) reflects the development of a ‘shared repertoire’ and the boundary-spanning practices required to connect CP@clinic with community organisations, health services and local communities. Theme 3 (data collection) illustrated Wenger’s concept of ‘reification’ whereby implementation experiences, service activities and outcomes were translated into data artefacts that could support reflection, evaluation and advocacy. Theme 4 (sustaining program implementation) reflects ‘shared practice’ and longer-term system alignment as participants worked collectively to embed CP@clinic within local service contexts and broader implementation priorities [18].

Within the CoP, mitigatinging program implementation challenges became a collective endeavour. For example, when community paramedics were challenged with data collection, through shared problem-solving, members developed practical strategies such as assessment templates, adapted training resources and documentation workarounds, while clarifying which clinical data were essential. This reflects Wenger’s participation-reification dynamic, where shared artefacts stabilise practice while allowing for local adaptation [19]. Importantly, the CoP also supported sensemaking about the purpose of data collection linking evaluation to learning, advocacy and funding rather than compliance alone. This reframing strengthened engagement with evaluation processes and aligns with implementation literature emphasising shared understanding of outcomes as key to sustainability [27].

Sustaining implementation further highlighted the limits of what a CoP can address independently. While the CoP enhanced continuity during staff turnover, maintained shared knowledge and strengthened program visibility and credibility, some barriers persisted beyond its scope. Funding insecurity, organisation commitment and digital infrastructure constraints represent out-setting determinants requiring system-level responses, rather than peer learning alone [28, 29]. These findings underscore that CoPs are powerful enablers within, but not substitutes for supportive, structural conditions.

### How the CoP supported program implementation

The CoP was central to supporting CP@clinic implementation by enabling relational learning, shared problem-solving and collective sensemaking across workforce development, community engagement and data collection [18]. Workforce development benefited from peer learning as the CoP provided a supportive environment where community paramedics could articulate uncertainties, validate emerging role identities and learn from colleagues navigating similar challenges. This helped ease the transition from emergency response to preventative, community-focussed primary care that aligns with Wenger’s conceptualisation of CoPs as spaces where knowledge and professional identity are co-constructed through participation [30]. Early role ambiguity observed in this study is consistent with evidence on paramedics entering primary care roles where expectations are negotiated across clinicians, patients and employers [26].

By viewing program implementation through the lens of the CoP, a suite of practical tools and networks were identified to support implementers with the practical *how* of implementation. Regular meetings, informal mentorship and opportunities for observation and shadowing reduced reliance on individual trial-and-error and helped normalise the steep learning curve associated with establishing clinics. These shared learnings built competence in assessment, referral navigation and documentation, reflecting broader evidence that CoPs facilitate practice improvement through mentorship and collaborative learning when appropriately supported [16, 31]. Community engagement was strengthened as participants shared local adaption strategies for building trust and tailoring activities to their community needs. These shared adaptations supported stronger relationships with community members and services, contributing to improved uptake. This aligns with rural primary healthcare literature emphasising that effective models are relationship-based, context sensitive and adaptable [32]. The CoP functioned as a site of joint enterprise and boundary work, where members collaboratively refined engagement approaches and shared locally adaptable knowledge rather than prescriptive guidance [19]. When mapping the CoP attributes (see Table 2) its utility in program implementation is evident [32, 33].

**Table 2:** Map of CoP attributes [18, 33] and findings

CoP attributes	Our results – examples	Associated theme
Mutual Engagement	Peer support Shadowing/mentoring	Workforce development
Problem solving	Data collection Advocacy for funding	Data collection Program sustainability
Sharing knowledge	Printing guides	Workforce development
Learning as social participation	Reflective discussions	Workforce development
Creating new understanding	Adapting the program	Program sustainability
Community	Engaging with stakeholders, referral pathways	Community engagement
Practice based identity	Transitioning from ambulance health to primary care	Workforce development
Knowledge transfer	Sharing from Canadians	Community engagement
Resources	Database management	Data collection

Methodologically, our analysis illustrates how the CoP operated simultaneously as an implementation strategy and as a source of evaluation insights. By examining CoP meeting records, we were able to elevate participant’s sensemaking of CP@clinic program implementation, negotiated role boundaries and professional identity making in real time. This underscores the utility of CoPs to contribute to both practice improvement and knowledge production for program implementation processes.

### Why the CoP matters

Beyond supporting the practical *what* and *how* of CP@clinic implementation, the CoP fulfilled a critical *why* function by providing a psychologically safe space in which uncertainly, vulnerability and ambiguity could be openly acknowledged. Participants described the CoP as a trusted environment where challenges could be discussed without judgement, enabling honest reflection on the clinical, professional and organisational tensions associated with implementing a novel model of care in rural contexts. This was particularly important for community paramedics navigating role transition, evolving scope of practice and professional identity alongside geographically dispersed and resource-constrained service delivery.

The CoP enabled collective sensemaking by reframing implementation challenges as expected features of innovation rather than individual or local failures. Through shared reflection and dialogue, participants transformed personal concerns into collective problems supporting resilience, confidence and sustained engagement. In this way, the CoP functioned not only as a site of knowledge exchange, but as social infrastructure that held the emotional and cognitive labour of implementation.

Importantly the CoP also supported meaning-making by linking everyday practice to a shared purpose centred on preventative care, equity and rural health system strengthening. This shared purpose helped sustain motivation despite ongoing structural constraints such as funding insecurity and workforce pressures. Consistent with Wenger’s conceptualisation of CoPs, these findings demonstrate that learning, identity and practice were co-constructed through participation over time. In the context of CP@clinic, the CoP matters because it created the relational conditions necessary for adaptation, confidence and continuity - conditions that technical solutions alone cannot provide.

### Limitations

This study draws on a small sample of self-reported CoP meeting data which may introduce bias and limit capture of dissenting perspectives. The data reflects internal discussions from program implementers, community paramedics and researchers; while acknowledging the absence of clients, other health professionals, policy makers and funders. The focus on early implementation stages constrains generalisability to longer-term outcomes and other healthcare settings. Findings may also have limited transferability beyond rural Australian contexts, where healthcare systems and community dynamics differ.

## Conclusion

This study examined the enablers and barriers to CP@clinic program implementation as identified through the CoP and how the CoP supported implementation in rural Australian contexts. Implementation was shaped by interconnected challenges related to workforce development, community engagement, data collection and program sustainability, including role uncertainty, the time required to build community trust, administrative and digital infrastructure constraints and concerns regarding funding continuity and workforce turnover.

The CoP emerged as a key enabler across all implementation domains. Through peer learning, shared problem solving and collective sensemaking, the CoP supported professional identity development, enabled locally responsive community engagement, strengthened data practices and provided continuity during organisational change. These findings position the CoP as a critical implementation mechanism supporting adaptive, contextually appropriate delivery of community paramedicine models such as CP@clinic, with demonstrated utility for program administrators and community paramedics implementing novel workforce models in rural health settings.

Future research should build on these findings by examining the impact of CP@clinic on health outcomes, emergency department utilisation, health system costs and workforce capability in the Australian context. Further, consideration needs to include client and external stakeholder perspectives to inform community paramedicine policy development. Combining outcomes‑focused evaluations with continued qualitative inquiry such as this study would provide a more comprehensive evidence base for embedding community paramedicine models within primary health care reform and for designing policy and funding frameworks that support their sustainability.

## Supplementary Information

Supplementary Material 1.

## Data Availability

The datasets generated supporting the findings of this study are not publicly available. Community of Practice meeting minutes contain sensitive contextual information and were collected under conditions of confidentiality, meaning public data sharing is not possible. De-identified extracts relevant to the study findings may be made available by the corresponding author on reasonable request, subject to ethics approval.

## Abbreviations

CoP: Community of Practice
CP@clinic: Community Paramedicine at Clinic
