# Supplementary material for: Paramedics in an emerging role; a reflexive thematic analysis of barriers and enablers to primary care innovation through a community of practice
Source: BMC Prim Care. 2026 Jun 23;27:319. doi: 10.1186/s12875-026-03426-y (PMC13520240; doi:10.1186/s12875-026-03426-y)
Supplement: Supplementary file 1 [file 12875_2026_3426_MOESM1_ESM.docx]

**Supplementary Material**

For: *Paramedics in an emerging role; a reflexive thematic analysis of barriers and enablers to primary care innovation through a Community of Practice*

**Researcher ‘behind the scenes’ statement and positionality**

As registered paramedics, FA, 5A and LA have a combined work experience of over 70 years nationally and internationally. This informed the research knowingness of the nature of the work, the workers and the workplace. From the commencement of the Australian-based implementation, 2A, 3A, 4A and 6A have been involved in research design of the pilot trial and subsequent scaled expansion.

**Supplementary Table 1: CoP meeting focus areas**

| Meeting # | Discussion focus |
| --- | --- |
| 1 | Overview of pilot, workforce utilisation, barriers and best practices |
| 2 | Referral pathways: challenges, enablers and workforce recruitment |
| 3 | Data collection challenges, risk factor discussions and capturing client engagement |
| 4 | Safety in community paramedicine: staff and client safety, job satisfaction and well-being |
| 5 | Workforce logistics: expansion, managing demand, service delivery and integration with services |
| 6 | Continuity of care in chronic disease self-management education |
| 7 | Communicating the role and benefits of community paramedicine |
| 8 | The emerging professional identity of community paramedics |
| 9 | Defining and measuring success in community paramedicine program delivery |
| 10 | Reflecting on end of 2024: program, practice and personal wins; data and advocacy |
| 11 | Program updates, data collection, funding, research and future directions |
| 12 | Implementation experiences, celebrating milestones, challenges and case studies |
| 13 | Community engagement, early learnings and collaboration opportunities |

**Supplementary Table 2: Attendance**

| Meeting | Attended | Attendance % |
| --- | --- | --- |
| 1 | 15/15 | 100% |
| 2 | 13/15 | 87% |
| 3 | No attendance recorded | N/A |
| 4 | 12/15 | 80% |
| 5 | 11/15 | 73% |
| 6 | 11/15 | 73% |
| 7 | 9/15 | 60% |
| 8* | 18/25 | 72% |
| 9 | 14/25 | 56% |
| 10* | 16/27 | 60% |
| 11 | No attendance recorded | N/A |
| 12 | 16/27 | 60% |
| 13 | 17/27 | 63% |

*Increased membership due to new joining programs

The total membership of the CoP increased over time from an initial 15 members in Meeting 1 to 27 members by Meeting 13. The increase in membership reflected the staged scaled implementation of additional sites and therefore inclusion of personnel. However, attendance at individual meetings remained relatively stable ranging from 9-18 attendees with a mean of 13.8 and median of 14 attendees per meeting. As membership expanded, attendance represented a smaller proportion of the total CoP membership and growing CoP. This pattern suggests that participation was characterised by a stable level of meeting engagement within an expanding community, comprising a core group of six (6) members regularly attending alongside additional rotating members who attended according to role, site involvement, operational demand and availability.

**The numerical counts presented in Supplementary Tables S3 and S4 are provided as descriptive context only; decisions about the importance of codes and themes were driven by their interpretive richness and contribution to the overall analytic story, rather than by frequency.

**Supplementary Table 3: Initial codes description**

| **Initial Codes** | **No of Meetings** | **No of Mentions** |
| --- | --- | --- |
| Financial | 4 | 8 |
| Social Connectedness | 4 | 12 |
| Identity Negotiation | 3 | 13 |
| Safety | 4 | 14 |
| Recruitment | 6 | 16 |
| Mentoring | 8 | 18 |
| Knowledge Transfer (Tacit) | 8 | 20 |
| Rural Communities or Ecosystem | 7 | 21 |
| Role Identity or Building | 6 | 21 |
| Evidence and Data Collection | 7 | 30 |
| Reflection of Practice | 12 | 39 |
| Problem Solving | 8 | 39 |
| Generating Ideas and Opportunities | 10 | 53 |

**Supplementary Table 4: Preliminary themes description**

| **Preliminary Themes** | **No of Meetings** | **No of Mentions** |
| --- | --- | --- |
| Safety | 4 | 14 |
| Evidence and Data Collection | 7 | 30 |
| Mentoring | 8 | 34 |
| Problem Solving | 8 | 39 |
| Rural Communities or Ecosystem | 7 | 53 |
| Financial | 4 | 61 |
| Role Identity or Building | 6 | 73 |

During analysis we moved iteratively from a larger set of preliminary themes (Supplementary Table 4) to the four final themes reported in the main paper: *workforce development, community engagement, data collection* and *sustaining program implementation*. Rather than treating each preliminary theme as a discrete endpoint, we examined how they clustered conceptually and how they contributed to an overarching interpretive account of CP@clinic implementation.

Several preliminary themes were subsumed within broader, more analytically coherent themes. For example, “mentoring”, “recruitment”, “role identity or building”, “knowledge transfer (tacit)” and “reflection of practice” consistently appeared in relation to paramedics’ transition into, and enactment of, the community paramedic role, so these were brought together within the final theme *workforce development*. Similarly, material coded to “rural communities or ecosystem”, “social connectedness” and “generating ideas and opportunities” was reconfigured within *community engagement*, which captured context-specific relationship-building, local partnerships and efforts to align the program with community needs.

“Evidence and data collection” remained a distinct analytic focus and was developed into the final theme *data collection*, while “financial” content was interpreted alongside broader discussions of funding stability, program visibility and system-level constraints to form *sustaining program implementation*. In keeping with reflexive thematic analysis, these decisions were guided by interpretive richness, coherence and contribution to the overall story of implementation, rather than by preliminary frequency counts or an expectation that each early theme would map directly onto a final theme.

**Supplementary Figure 1: CoP meetings - NVivo codes merged into final themes**

**Supplementary Table 5: Reflexive thematic steps** (21, 24)

| **Step** | **Description** | **Application to data** |
| --- | --- | --- |
| Meeting #1 | | |
| 1. Familiarisation | FA and LA immersed into the reading and re-reading meeting records to identify key points. | Reviewing discussions across multiple meetings to understand recurring issues. |
| 2. Coding | Systematically coding relevant features of the data across the dataset. | Coding topics such as "data collection challenges," "community engagement strategies," and "role transition reflections". |
| Meeting #2 | | |
| 3. Generating initial themes | Collating codes into potential themes based on patterns observed in the data. | Grouping codes into broader categories. |
| 4. Developing and reviewing themes | Refining themes by checking against the dataset for coherence and relevance. | Ensuring themes align with the meeting content. |
| Meeting #3 | | |
| 5. Refining, defining and Naming Themes | Clearly defining each theme and determining its scope. | Naming themes while specifying their focus. |
| 6. Writing up | Writing up the themes with supporting examples from the data. | Creating tables summarising themes with direct references to meeting records. |
| Meeting #4 | | |

**Supplementary Table 6: Themes with descriptors and mentions**

| **Final Theme** | **Description** | **Total No of Mentions*** |
| --- | --- | --- |
| Data Collection | Emphasis on collecting robust data to demonstrate program impact and secure funding | 30 |
| Community Engagement | Importance of building relationships with local communities to improve healthcare access and outcomes | 53 |
| Sustaining Program Implementation | Strategies to ensure long-term funding and integration into healthcare systems | 100 |
| Workforce Development | Challenges and strategies for recruiting, training and retaining Community Paramedics | 121 |

*Approximate counts are provided only; theme salience and inclusion were determined by interpretative richness and analytic contribution, not by frequency.

**Supplementary Figure 2: Theme 1 - Workforce development**

***Supplemental Note 1 (SN1) Theme 1 - Workforce development***

***Barriers and challenges* (*the what*)**

Participants described workforce development as a significant challenge during CP@clinic program implementation particularly in relation to role clarity, training and transitioning from ambulance-based practice to community and primary care settings. Several participants highlighted the steep learning curve associated with entering the community paramedic role, especially when training, service delivery and clinic establishment occurred simultaneously.

***CoP support for implementation (the how)***

The CoP supported workforce development by enabling shared learning, mentorship and peer support. The value of these approaches was reinforced by participants who reported that shadowing existing staff was beneficial during their transition into the role.

Participants described significant ambiguity around the community paramedic role. Recruitment was complicated by inconsistent expectations across regions and newly appointed community paramedics often lacked clarity around the preventative and relational nature of the role. In Meeting #1 it was identified that *‘…barriers included unfamiliarity with the role outside of the ambulance service, lack of professional development and no defined salary structure.’* However, in addressing these barriers *‘…learning opportunities in health coaching and navigating aged care, which were new to them as a paramedic’* were identified.

The CoP supported workforce development by enabling shared learning and co-construction of the role. Paramedics used the CoP to validate their experiences, clarify role expectations and develop practical responses to common challenges. In Meeting #1 it was noted that ‘… *the community paramedic role requires a different way of thinking for newcomers’* and that mentorship *‘… was valuable in guiding their transition.’* Differences from ‘ambulance health’ practice were also identified such as *‘…building rapport with clients, a departure from the emergency sector where relationships with clients are minimal.’*

**Supplementary Figure 3: Theme 2 - Community engagement**

***Supplemental Note 2 (SN2) Theme 2 - Community engagement***

***Barriers and challenges (the what)***

Gaining trust and visibility in local communities was both essential and complex. Participants noted that success depended heavily on context-specific engagement strategies, relationships with local health partners and the ability to align CP@clinic services with community-identified needs. In Meeting #9 it was acknowledged that meaningful consumer engagement was critical to the program, while barriers were identified, particularly among consumers with low technology literacy. Consumer engagement was attributed to program ‘*early wins’*, including improved access to healthcare, successful patient referrals and increased community trust (Meeting #11).

Consumer engagement was described as difficult in some contexts particularly where technology literacy was low. Participants also identified the need to build relationships with primary care providers, noting that *‘…GPs and NPs (nurse practitioners) within the service [are] important to establish relationships and referral pathways,’* and that *‘…being able to build relationships in order to have priority referral pathways so CP@clinic clients don’t have to wait’* is helpful.

***CoP support for implementation (the how)***

The CoP discussions provided a reflective space for implementers to share and adapt community-specific strategies and engagement techniques. Implementers used these discussions to troubleshoot low attendance, develop outreach plans and build a deeper understanding of local dynamics.

Implementers shared practical strategies, adapt approaches and built local partnerships (Meeting #9). Participants described service development through collaboration with local organisations, noting that ‘…*discussions are underway with service providers such as (name of service) to host clients in their facilities, with a focus on existing sites and additional locations based on community needs.’* Workforce and service planning were similarly framed around community need, with meeting notes stating that ‘the selected recruit will work alongside community health nurses, initially targeting areas identified as underserved with significant healthcare needs.’

Participants described strategies for increasing visibility and community participation including events and outreach activities. For example, Meeting #13 noted *‘… the first two clinics, which included community barbecues where they engaged with clients and conducted health assessments.’* Other approaches included targeted promotion and local tailoring with participants sharing *‘…plans to enhance community engagement at (clinic location) by promoting sessions to local groups and incorporating guest speakers such as dietitians to address specific health concerns like high cholesterol’* (Meeting #13). Participants also discussed what worked in attracting attendance including *‘…suggestions for collaborating with communities - what works (key neighbourhood ‘influencers’ and food)’* and what does not such as, ‘…*sale yards are ‘work’* with one participant reflecting on the significance of food in drawing people together (Meeting #13).

In supporting engagement through shared learning about referral pathways and service coordination, participants described strategies such as *‘…doing warm handovers to familiarise yourself with the local services - and spend some time with those services to understand how they operate (and for them to understand what you do)’* (Meeting #2). Participants described engagement outcomes and early indicators of impact, noting *‘early wins including improved access to healthcare, successful patient referrals and increased community trust’* and that *‘many participants in the program are disengaged from traditional healthcare, but the program is successfully re-engaging them through accessible services and additional support like food relief and community meals.’* The role of accessible service design was also emphasised with clinics described as *‘.... free, located in community centres and retirement villages, with no appointments required’* (Meeting #7).

**Supplementary Figure 4: Theme 3 - Data collection**

***Supplemental Note 3 (SN3) Theme 3 - Data collection***

***Barriers and challenges (the what)***

Data capture for evaluation and reporting purposes presented practical challenges, particularly in rural areas with limited infrastructure. Participants described tensions between service delivery demands and administrative workload at times.

Participants consistently identified data collection as a practical challenge during CP@clinic implementation. Administrative burden, technological limitations and the complexity of managing multiple documentation systems were frequently discussed. Meeting notes acknowledged that *‘the extensive data collection requirements could be overwhelming for paramedics*,’ particularly for new clinics navigating systems for the first time.

**Supplementary Figure 5: Theme 4 - Program sustainability**

***Supplemental Note 4 (SN4) Theme 4 - Sustaining program implementation***

***Barriers and challenges (the what)***

Participants expressed concerns about long-term sustainability due to funding uncertainty leading to increased staff turnover. The CoP was consistently identified as a stabilising factor that supported continuity, accountability and adaptive problem solving. Participants viewed the CoP as central to sustaining implementation momentum over time, highlighting *‘…the value of CoP as a space for reflection and peer support’ (Meeting #4).* It was clarified that the CoP *‘… reinforced* *that the data gathered would not only demonstrate effectiveness but also support strategic arguments to secure funding for expanding paramedics’ roles in primary care’* (Meeting #9).

A recurrent concern expressed across CoP discussions was the challenge of sustaining CP@clinic over time. Funding uncertainty was repeatedly identified as a key barrier, contributing to anxiety about workforce stability and long-term service continuity. It was emphasised that ‘… *the need for funding certainty and longer-term assurance to continue building the program’s momentum*.’ Another participant similarly described ongoing pressure to demonstrate success, noting ‘*there is always a worry that if the service isn’t ‘successful’ then funding will be discontinued and ultimately, clients will be left without help*.’

Participants also identified broader system level constraints affecting sustainability. These included challenges related to legislation, employer practices, funding models, education and technology as highlighted during discussions of the national scope of practice review. Workforce wellbeing was raised as an additional concern, with meeting notes indicating ‘*the importance of staff wellbeing and the sustainability of community health programs.*’

***CoP support for implementation (the how)***

The CoP was consistently described as a stabilising mechanism that supported continuity, shared accountability and adaptive problem-solving. Recruitment and succession planning were also discussed within the CoP, including updates on interviews, funding arrangements and collaborative training plans to support transitions for new staff.
